# Naturally Occurring Deletion Mutants of the Pig-Specific, Intestinal Crypt Epithelial Cell Protein *CLCA4b* without Apparent Phenotype

**DOI:** 10.1371/journal.pone.0140050

**Published:** 2015-10-16

**Authors:** Stephanie Plog, Nikolai Klymiuk, Stefanie Binder, Matthew J. Van Hook, Wallace B. Thoreson, Achim D. Gruber, Lars Mundhenk

**Affiliations:** 1 Department of Veterinary Pathology, Faculty of Veterinary Medicine, Freie Universität Berlin, Berlin, Germany; 2 Institute of Molecular Animal Breeding and Biotechnology, Ludwig-Maximilians-Universität, Munich, Oberschleissheim, Germany; 3 Department of Ophthalmology and Visual Sciences, University of Nebraska Medical Center, Omaha, Nebraska, United States of America; The Ohio State University, UNITED STATES

## Abstract

The human *CLCA4* (chloride channel regulator, calcium-activated) modulates the intestinal phenotype of cystic fibrosis (CF) patients via an as yet unknown pathway. With the generation of new porcine CF models, species-specific differences between human modifiers of CF and their porcine orthologs are considered critical for the translation of experimental data. Specifically, the porcine ortholog to the human CF modulator gene *CLCA4* has recently been shown to be duplicated into two separate genes, *CLCA4a* and *CLCA4b*. Here, we characterize the duplication product, *CLCA4b*, in terms of its genomic structure, tissue and cellular expression patterns as well as its *in vitro* electrophysiological properties. The *CLCA4b* gene is a pig-specific duplication product of the *CLCA4* ancestor and its protein is exclusively expressed in small and large intestinal crypt epithelial cells, a niche specifically occupied by no other porcine *CLCA* family member. Surprisingly, a unique deleterious mutation of the *CLCA4b* gene is spread among modern and ancient breeds in the pig population, but this mutation did not result in an apparent phenotype in homozygously affected animals. Electrophysiologically, neither the products of the wild type nor of the mutated *CLCA4b* genes were able to evoke a calcium-activated anion conductance, a consensus feature of other *CLCA* proteins. The apparently pig-specific duplication of the *CLCA4* gene with unique expression of the *CLCA4b* protein variant in intestinal crypt epithelial cells where the porcine CFTR is also present raises the question of whether it may modulate the porcine CF phenotype. Moreover, the naturally occurring null variant of *CLCA4b* will be valuable for the understanding of *CLCA* protein function and their relevance in modulating the CF phenotype.

## Introduction

Proteins of the chloride channel regulator, calcium activated (CLCA) family are putative modulators of the cystic fibrosis (CF, mucoviscidosis) phenotype, a lethal inherited disease caused by mutations in the *Cystic Fibrosis Transmembrane Conductance Regulator* (*CFTR*) gene [1–4]. These gene defects result in its functional loss as a chloride and bicarbonate channel, consequently leading to mucus plugging in various organs including the respiratory and intestinal tracts, with the hallmark lesion being a secondary bacterial lung infection [5]. The CF phenotype differs widely in severity between patients despite identical *CFTR* mutations. Disease outcome, quality and expectancy of life vary remarkably even between siblings bearing the same genetic defect [6,7]. In addition to environmental factors, various genetic modifiers of the disease have been suspected to account for this variability [7]. Among these putative modulators is an alternative chloride current which has been proposed to at least partially compensate for the loss of the CFTR-mediated chloride secretion in CF tissues [8–10].

The human CLCA members CLCA1 and CLCA4 are expressed in CF-relevant tissues and their allelic variants have been identified as modulators of the intestinal residual anion conductance in CF patients [1,3,4,11,12]. In contrast to earlier conceptions, the proteins do not form ion channels themselves but appear to modulate calcium-dependent chloride conductances probably as a modifier of TMEM16A activity [13–16].

The modulatory role of CLCA proteins has been confirmed in mouse models of CF. The intestinal phenotype of CF mice is ameliorated by experimental overexpression of Clca1 (formerly known as mClca3, now renamed according to nomenclature in humans) [2]. The murine Clca4a (formerly known as mClca6) is a known inducer of a calcium-activated chloride conductance and its protein co-localizes with that of the murine CFTR in enterocytes [17,18]. However, all murine models of CF have important drawbacks limiting their usefulness in translational research on CF and CLCA proteins. In terms of CF, none of the murine models available reflects the complexity of the human CF phenotype, primarily due to their lack of the characteristic respiratory pathology [19]. Furthermore, drastic differences exist between the human and murine *CLCA* gene loci, with four *CLCA* genes in humans and eight in mice [20]. Moreover, the human *CLCA3* is thought to represent a pseudogene, in contrast to its murine counterparts *Clca3a1*, -*3a2* and -*3b* (formerly known as *mClca1*, -*2*, -*4*) [21]. Another important difference is the variance between the cellular expression patterns of the murine Clca4a and its human ortholog CLCA4 [17].

In contrast to murine CF models, the newly generated CF pigs develop all hallmark features of the human disease including respiratory pathology with mucus overproduction and secondary bacterial pneumonia. Thus, CF pigs are expected to become the more relevant animal model in CF research [22–27].

Considering both the potential of the porcine CF model and the role of CLCA gene products in CF, we have started to characterize the CLCA gene family in the pig, particularly focusing on possible differences between pigs, humans, and mice [28–30]. Although our data have revealed essential similarities between porcine and human *CLCA* genes and their proteins, a species-specific gene duplication of the porcine *CLCA4* ortholog was found, resulting in *CLCA4a* and *CLCA4b* [28] with the former being recently characterized in detail [29].

Here, we describe genetic characteristics, the tissue and cellular expression patterns and the *in vitro* calcium-activated chloride conductance signature of CLCA4b. One entirely unexpected finding of this study was the discovery of naturally occurring *CLCA4b* gene silencing in a large subset of the porcine population, resulting in complete lack of the protein with no obvious phenotype.

## Material and Methods

### Genetic Characterization

Exon/intron structures, the coding region as well as the amino acid sequence of *CLCA4b* were identified using BioEdit as described, by using the porcine *CLCA4a*, the human *CLCA4* and the murine *Clca4a* as reference genes [28,31]. In order to identify potential regulatory properties of the *CLCA4b* upstream region, sequences between *CLCA4* genes and the upstream located gene *CLCA1* and downstream located *CLCA3* from 12 different mammalian species (human, macaque, marmoset, mouse, rat, cat, dog, panda, horse, cattle, alpaca, and dolphin) as well as the intergenic regions of porcine *CLCA1* and *CLCA4a*, *CLCA4a* and *CLCA4b* as well as *CLCA4b* and *CLCA3* were obtained from the ensemble database (www.ensembl.org) and aligned using Clustal Omega (http://www.ebi.ac.uk/Tools/msa/clustalo/). Potential conserved binding sites for transcription factors were determined by Genomatix ElDorado/Gene2Promoter and GEMS Launcher software packages (*Genomatix*, *Munich*, *Germany*), according to Wünsch and colleagues [32]. Repetitive elements identified by RepeatMasker (http://www.repeatmasker.org) were excluded from the analysis. Upstream regions of *CLCA4a* and *pCLCA4b* were compared to human and bovine genomic regions by BLASTn (http://blast.ncbi.nlm.nih.gov/Blast.cgi). To evaluate evolutionary forces on the *CLCA4* genes, the coding sequences from man, macaque, rat, mouse, horse, cat, dog, sheep, and cattle CLCA4 as well as those from porcine CLCA4a and CLCA4b were compared in a multiple sequence alignment. Conserved protein domains were identified by BLASTp (http://blast.ncbi.nlm.nih.gov/Blast.cgi) and putative sites prone to positive selection were identified by the codonML software from the PAML package (http://abacus.gene.ucl.ac.uk/software/paml.html). Phylogenetic trees were calculated as described [33].

### Animals, Tissue Processing and Ethical Statement

Tissues from six-week-old male EUROC x Pietrain pigs (n = 5 from one litter) as well as the genital tracts of two- or three- months-old female mixed breed pigs (n = 2) were used to study the expression pattern of *pCLCA4b* on RNA level using RT-PCR and on protein level using immunohistochemistry as described [29]. Mixed breed pigs were screened for the mutation on genomic level as described below. In addition, porcine samples of the colon and jejunum from pigs were snap frozen or fixed in 4% formalin for expression analyses on RNA and protein level, respectively. All experiments were approved according to the rules of the German Animal Welfare Law by the ethical committee of the local governmental authorities (State Office of Health and Social Affairs Berlin, approval IDs: O 0401/12 and T 0400/12). The pigs were housed in groups in the ruminant and swine clinic of the Veterinary Department of Freie Universität Berlin which is a veterinary surgery training institute approved by the European Association of Establishments for Veterinary Education (EAEVE). The surgery to neuter pigs was performed under ketamine and azaperone anesthesia, and all efforts were made to minimize suffering. Euthanasia, if applicable, was performed by intravenous or intracardial application of embutramide in deep ketamine/azaperone anesthesia. Surgeries and euthanasia which took place at the ruminant and swine clinic of the Veterinary Department of Freie Universität Berlin were conducted by a veterinarian.

### Genotyping of Animals

Genomic DNA was isolated from testicles obtained during neutering animals, from ear marking biopsies or skin from routinely autopsied pigs by addition of 100 μl isolation buffer (2 M Tris pH 8.5, 0.5 M EDTA, 10% SDS, 5 M NaCl) with 12 μl proteinase K (10mg/ml; Carl Roth GmbH & Co. KG, Karlsruhe, Germany). The samples were incubated at 55°C for 3 hrs, followed by incubation at 95°C for 10 min. Subsequently, 800 μl TE/RNase A mix (0.5 M EDTA pH 8.0, 1 M Tris pH 7.6, 10 mg/ml RNase A) were added to each sample. After a final heating at 99°C for 15 min, the samples were frozen at -20°C until further use. In addition, whole genomic DNA from ten different porcine breeds (Mangalitsa, Hampshire, Large White, Cerdo Iberico, Pietrain, Duroc, Swabian, Large Black, German Landrace, Turopolja; n = 3 to 5 per breed) were genotyped for the presence of the mutation. To distinguish between pigs carrying the mutation and wild type animals, a restriction endonuclease assay was established on a specific genomic DNA amplicon. In a first step, PCR products of the genomic DNA containing the deletion were amplified using primers 5’-ACACCTGATAAGTAATGCCCTGGA-3’ (upstream) and 5’-CCGCATTTGGCCCGAGAGCA-3’ (downstream) according to the following protocol: 30 cycles at 95°C for 2 min, 95°C for 30 sec, 64°C for 30 sec and 72°C for 15 sec with a final extension at 72°C for 10 min. Second, *Dra*I (FastDigest, Fermentas, St. Leon-Rot), recognizing the DNA site 5'-…TTT↓AAA…-3', cleaved the amplicon of genomic CLCA4b DNA only in case of the genomic deletion. In contrast, *Tsp*509I (FastDigest, Fermentas, St. Leon-Rot), cutting at 5'-…↓AATT…-3', only processed the amplicon of non-mutated genomic CLCA4b DNA. Each of the samples was additionally tested using primers discriminating mutated genomic DNA against wild type genomic DNA (Table 1) and / or complete sequencing.

**Table 1:** Primers used for distinction between *CLCA4b* genomic DNA with and without the 10 base pair deletion.

Primer name	Sequence
p4b-ND-For	5’-CTGGATTATGTTTGCAGGTAATTAAAG-3’
p4b-ND-Rev	5’-GTTACGGCGCTCATTTCTGT-3’
p4b-D-For	5’-GCCCTGGATTATGTTTAAAG-3’
p4b-D-Rev	5’-GTTACGGCGCTCATTTCTGT-3’

ND primers used for amplification of genomic *CLCA4b* without sequence deletion with an amplicon length of 192 bp. Protocol: 95C/ 2 min, 95C / 30 sec, 60C /30 sec, 72C / 6 sec, 72C / 10 min, 30 cycles

D primers used for amplification of genomic *CLCA4b* with sequence deletion with an amplicon length of 186 bp. Protocol: 95C / 2 min, 95C / 30 sec, 60C / 30 sec, 72C / 6 sec, 72C / 10 min, 30 cycles

### Cloning and Sequencing of the *CLCA4b* Gene

Total RNA isolated from the colon of three different pigs was reverse transcribed with SuperScript III First-Strand Synthesis System (Invitrogen, Karlsruhe, Germany) as described [28]. The open reading frame (ORF) of *CLCA4b* (Plog et al. 2009; GenBank accession nos. XM_003125934.2 and CU469041.5) was amplified using the primers 5’-GAAAAGCCTCTTGAACAAG-3’ (upstream) and 5’-ACCTAAATATCCATTCTAGATT-3’ (downstream) and High Fidelity Taq polymerase (Fermentas, St. Leon-Rot, Germany) as follows: 30 cycles at 94°C for 3 min, 94°C for 30 sec, 49.3°C for 30 sec, 72°C for 3 min, 72°C for 10 min with a time increment of 2 sec per cycle. The coding sequences with and without the deleterious mutation were cloned into pcDNA3.1 by T-addition as described [28]. With exception of the mutation, 100% sequence identity was confirmed via complete sequencing. In addition, the amplified cDNA of three different pigs bearing the mutation was sequenced for verification purposes without subsequent cloning.

### Sequence Characterization and Generation of Antibodies

*In silico* analyses of the pCLCA4b amino acid sequence and the generation of antisera against pCLCA4b were performed as described [28,29]. In brief, rabbits were immunized with the following oligopeptides located within the amino-terminus of the pCLCA4b protein (p4b-N-1; HFYTTDQSESRGLT; corresponding to amino acid positions aa 455–468, and p4b-N-2; KLIQIKSNNERRKL; corresponding to amino acids 356–369). The obtained antisera were tested via immunoblotting for specificity as described below, and serum p4b-N-1 was found most suitable for immunoblotting and immunohistochemistry whereas p4b-N-2 only succeeded in immunoblotting experiments using CLCA4b-transfected cells.

### Transient Transfection of HEK293 Cells and Immunoblotting

HEK293 cells were transiently transfected with the CLCA4b ORF cloned into pcDNA3.1 and processed for immunoblotting as described [28,29]. Snap frozen whole tissue sections or scraped mucosa of small and large intestine were homogenized using a Precellys 24 (peqlab Biotechnologie GmbH, Erlangen, Germany).

Immunoblot analyses were performed as described using the anti-pCLCA4b antibodies p4b-N-1 (1:5,000) or p4b-N-2 (1:5,000) [28]. The corresponding preimmune sera (1:5,000) or absorbed p4b-N-1 serum (with 20 μg/ml of its specific peptide or with an irrelevant peptide) served as negative controls.

### mRNA and Protein Expression Patterns in Tissues

Total RNA was isolated as described [27]. Specific CLCA4b primers were designed using Primer BLAST (http://www.ncbi.nlm.nih.gov/tools/primer-blast/) to amplify a 172 base pairs product (5’-CAGGCTGCTGGAAAAGCAAAAACTG-3’; 5’-CAGACGCTATTCTGGTGAAACTCTC-3’). PCR amplification using DreamTaq DNA Polymerase (Fermentas, St. Leon-Rot, Germany) included 34 cycles at 95°C for 2 min, 95°C for 30 sec, 70.5°C for 30 sec and 72°C for 30 sec with a final extension at 72°C for 10 min. Representative amplicons from small intestine, large intestine and nasal mucosa were additionally sequenced and tested via BLASTn (http://blast.ncbi.nlm.nih.gov/Blast.cgi) to confirm 100% sequence identity with CLCA4b. All experiments were repeated at least twice. The mRNA quality and efficacy of reverse transcription were tested as described [29]. To exclude cross reactivity with any of the other porcine CLCA members, PCR reactions were carried out using the cloned ORFs of CLCA1 [28], CLCA2 [30], or CLCA4a [29] as templates. cDNA of jejunal or colonic samples from homozygous wild type (n = 3), homozygous mutated (n = 5) or heterozygous (n = 4) pigs was subsequently used as template in a quantitative RT-PCR analysis using Maxima Probe qPCRMasterMix (Fermentas). Primers, taqman probes and protocols are listed in Table 2. Target gene expression was quantified as described [17].

**Table 2:** Primers used for quantitative RT-PCR investigation.

Primer & probes	Sequence
p4b-For-qPCR	5’-AGTGAAACAAAGTGGGGCCATC-3’
p4b-Rev-qPCR	5’-GAAATGCATCCCTCCTGTTACGG-3’
p4b taqman probe	5’-CTATTGCTCTCGGGCCAAATGCGGACC-3’
EF-1a-For-qPCR	5’-AGAACATGATTACAGGCACTTCCC-3’
EF-1a-Rev-qPCR	5’-GGAAATACCTGCTTCGAATTCACC-3’
EF-1a taqman probe	5’-ACCAGCAGCAACAATCAGGACAGCACA-3’

p4b primer & probes: Amplicon length100 bp. Protocol: 95C / 3 min, 95C / 10 sec, 61,4C / 30 sec, 40 cycles

EF-1a primer & probes: Amplicon length 86 bp. Protocol: 95C / 3 min, 95C / 10 sec, 61.4C / 30 sec, 40 cycles

For immunohistochemical protein localization in cells and tissues, formalin-fixed, paraffin-embedded tissues were processed as described [33] using anti-CLCA4b antibody p4b-N-1, pre-immune sera or the antibody preincubated with 50 μg/ml of its specific peptide. Antibody dilutions ranged from 1:500 to 1:10,000 with optimal results at 1:8,000. In addition, consecutive slides were stained with specific antibodies against CLCA1 [28] or CLCA2 [30] in mutated and wild type pigs, respectively. Cryostat sections were used for expression analysis of CLCA4a [29]. Consecutive sections were also analyzed histopathologically using standard hematoxylin & eosin (H&E) staining.

### *In vitro* Electrophysiology

*In vitro* electrophysiological assays were conducted as described [34] with the equine CLCA1 clone as positive control for induction of a calcium-activated chloride current. The cDNA of the two different *CLCA4b* clones (*CLCA4b* wt, *CLCA4b* mut) or *CLCA1* was co-transfected with EGFP or RFP cDNA into HEK293 cells (American Type Culture Collection, Manassas, VA USA) using Lipofectamine 2000 Transfection Reagent (Invitrogen, Carlsbad, CA USA). CLCA-expressing cells were targeted for whole-cell recordings based on EGFP or RFP fluorescence 12–24 hours after transfection. Whole cell recordings were obtained using patch electrodes pulled from borosilicate glass (1.2 mm OD, 0.95 mm ID, with an internal filament) with a Narishige PC-10 vertical pipette puller. The resistance was 8–12 MΩ when the pipettes were filled with an internal solution containing (in mM) 98 KCH_3_SO_4_, 44 KCl, 3 NaCl, 3 MgCl_2_, 1 CaCl_2_, 3 EGTA, 5 HEPES, 2 glucose, 1 Mg-ATP, 1 GTP-Na, 1 reduced glutathione (pH = 7.8, osmolarity = 287 mOsm). Cells were constantly superfused at ~1 ml/min with an oxygenated extracellular solution containing (in mM) 140 NaCl, 5 KCl, 2 CaCl_2_, 1 MgCl_2_, 10 HEPES, 10 glucose (pH = 7.4, osmolarity = 290–295 mOsm) and voltage-clamped at -50 mV using an Axopatch 200B amplifier (Axon Instruments/Molecular Devices; Sunnyvale, CA). Test pulses were delivered and currents were acquired using pClamp 9.2 with a Digidata 1322 interface (Axon Instruments/Molecular Devices). Ionomycin, a calcium ionophore used to stimulate an increase in intracellular [Ca^2+^], was diluted 1:1,000 to an extracellular working solution concentration of 10 μM from a stock prepared in DMSO. The Ca^2+^-evoked current-voltage (I-V) relationship was measured as the difference of the currents recorded in response to a series of voltage steps (150 ms, -110 to -10 mV) before and after ionomycin application. The reversal potential of the ionomycin-evoked current was determined by linear extrapolation. Experiments were conducted at room temperature. With these recording solutions, E_Cl_ was predicted to be -25 mV. All chemicals were obtained from Sigma-Aldrich (St. Louis, MO, USA) except for KCH_3_SO_4_ which was obtained from Pfaltz and Bauer (Waterbury, CT, USA) and ionomycin which was obtained from Tocris (Ellisville, MO USA).

### Statistical Analysis

All data are presented as mean ± SEM. Statistical comparisons were performed using Student’s t-test. A P value of < 0.05 was considered to be statistically significant.

## Results

### Porcine *CLCA4b* is a unique duplication of *CLCA4a* and a naturally occurring deletion mutant of *CLCA4b* exists in the porcine population

**Fig 1:**
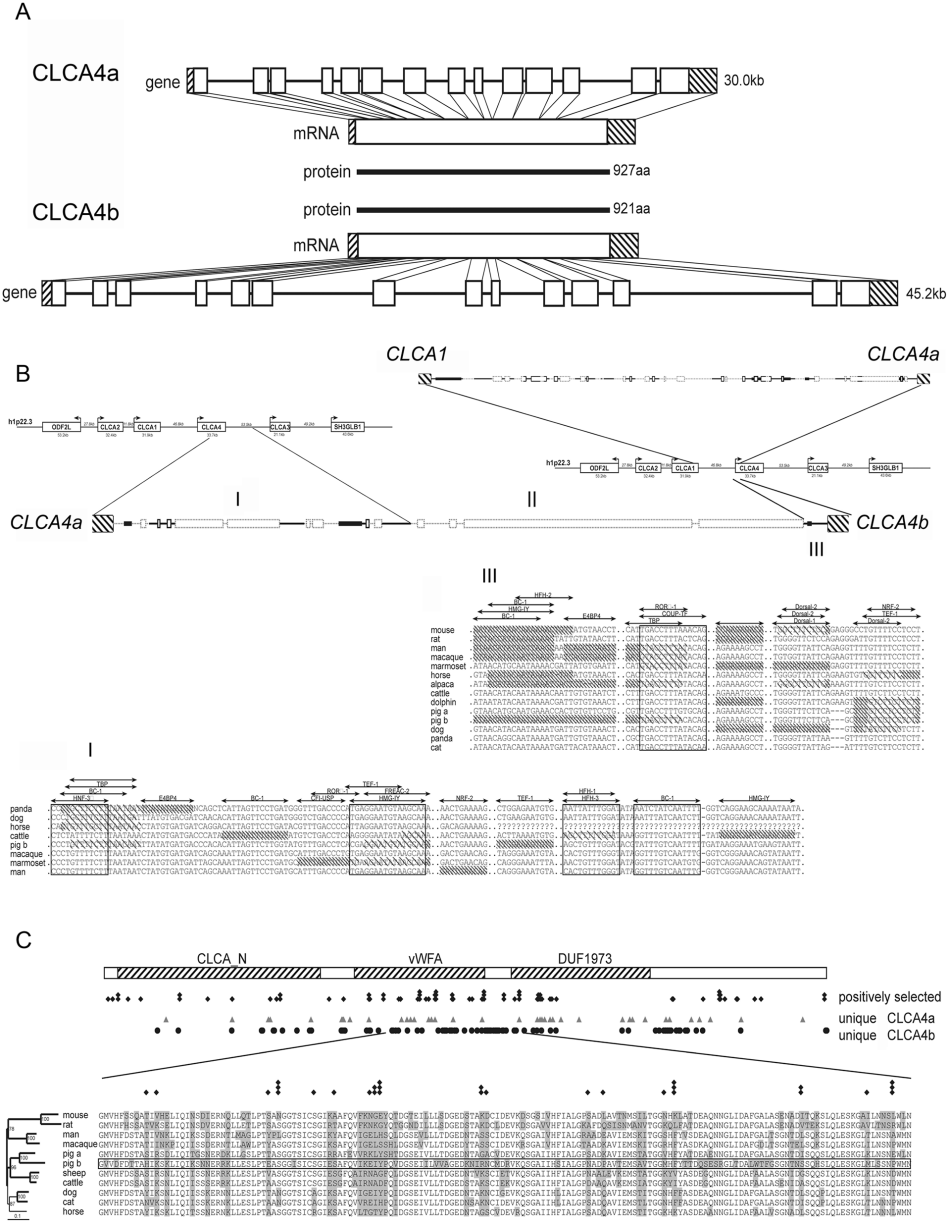
Comparison of the genomic structures of *CLCA4a* and *CLCA4b*, their promoter regions and the encoded proteins sequences. (A) Albeit having longer intronic sequences than *CLCA4a* (top panel), the genomic structure of *CLCA4b* (bottom panel) was highly similar to its paralog with the same number of exons (boxes) and has an open reading frame according to its ortholog. Due to a deletion of one amino acid in exon 2 and a gap of 5 amino acids in exon 14 the translated CLCA4b protein is slightly shorter than the CLCA4a protein with 921 vs. 927 amino acids (aa). (B) The intergenic region between *CLCA1* and *CLCA4a* showed high homology to the orthologous bovine and human regions (top panel). In the intergenic region between *CLCA4a* and *4b*, only the sequence downstream to *CLCA4a* showed homology to the corresponding bovine and human regions (I) followed by a sequence with no similarity to any sequence of mammalian CLCA loci investigated (II). In contrast, the region proximal to *CLCA4b* had homology to the intergenic region upstream from the human and bovine *CLCA4* gene (III) (bold style: regions with similarity to both species, faint style: regions without similarity to any of the species, boxes: repetitive elements). In regions I and III, parts are conserved among the examined mammalian species and contain numerous putative transcription factor binding sites (TFB). Sequences not fully conserved at TFB sites in distinct species are shaded; sequences not matching the prerequisites of the binding sites in distinct species are densely shaded. C) Alignment of 11 protein sequences clustering to the CLCA4 family revealed three conserved domains, the CLCA_N domain, the van Willebrand-factor A domain and a domain of unknown function (DUF). Sites of positive selection were defined by the codonML software, using the model algorithms 3 (discrete, naïve empirical bayes) and 8 (beta & omega > 1, naïve empirical bayes). Sites that are proposed to be positively selected are marked with a rhombus, sites that are proposed as significantly (p > 95%) or highly significantly (p > 99%) positively selected in both algorithms have two or three rhombi, respectively. In comparison, positions that are unique for CLCA4a or CLCA4b are labeled with triangulars or circles, respectively. A conserved region covering parts of the vWFA domain as well as the downstream sequence is shown in detail. Of the 38 unique sites in pCLCA4b in this region, 15 are located at sites under putative positive selection.

The duplication of the *CLCA4* gene into *CLCA4a* and *CLCA4b* appears to be a unique event in the pig and has not been observed in any other species analyzed so far [28]. A systematic analysis revealed an overall highly similar genetic structure to *CLCA4a*, albeit with longer intron sequences (Fig 1A). Like *CLCA4a* and other mammalian *CLCA4* genes [29], *CLCA4b* is also composed of 14 exons and the protein is only six amino acids shorter than pCLCA4a, due to a single amino acid deletion in exon 2 and a second deletion of 5 amino acids in exon 14. To determine the evolutionary history of the porcine *CLCA4* duplication, the genomic region between *CLCA1* and *CLCA3* genes which contain *CLCA4a* and *CLCA4b* in the pig and *CLCA4* in all other examined mammalian species were compared (Fig 1B). Throughout the entire length of 29 kb, the region between *CLCA1* and *CLCA4a* showed high homology with the corresponding regions in humans and cattle. The downstream region of the *CLCA4a* gene showed similarity to the corresponding regions between mammalian *CLCA4* and *CLCA3* genes for 11.6 kb, but then in the pig a region follows that lack any similarity to other mammalian *CLCA* loci and rather comprises repetitive elements. Further downstream of porcine *CLCA4a*, only a short region of 0.5 kb, quite upstream of the *CLCA4b* transcription start site, showed similarity to the region upstream of the bovine *CLCA4*, suggesting that *CLCA4b* resulted from a duplication of an initial porcine *CLCA4* ancestor gene. Thus, while *CLCA4a* comprises the original gene with the entire regulatory elements of the ancestor, the regulatory properties of *CLCA4b* might have been altered as a result of the duplication. Although we have found numerous potential binding sites for transcription factors (TFB) in the upstream region of *pCLCA4b*, the length of this potential proximal promoter is much shorter than the conserved putative promoters of other mammalian *CLCA4*, including porcine *CLCA4a*. On the other hand, putative unique enhancer elements of porcine *CLCA4b* were identified as conserved and potential regulatory elements that are otherwise located downstream of *CLCA4* in other mammalian species are positioned upstream of the porcine *CLCA4b* as a result of the gene duplication.

As the assembly of novel regulatory properties might have resulted in a speciation of the two porcine *CLCA4* genes, we evaluated whether a change in evolutionary pressure took place after gene duplication. Assuming that evolutionary adaptation may preferentially take place at sites prone to positive selection in the mammalian *CLCA4* genes, we analyzed the accumulation of unique sites in the CLCA4a and 4b protein in these regions (Fig 1C). Within the otherwise highly conserved N-domain, the van Willebrand-factor A domain and a domain of unknown function (DUF), we identified positions which may be under positive selection. Among those, a region of 160 amino acids, constituting a proportion of the van Willebrand-factor A domain and the adjacent downstream region, is highly conserved among the examined species. However, 18 positions yield evidence of positive selection. 38 amino acids within this stretch are unique for the pCLCA4b protein (23.7%) whereas the amount of unique amino acids in the other examined CLCA members is only between 3 and 21 (8.7 ± 5.6). Additionally, of the 18 positions under positive selection, 15 show differences between the CLCA4a and CLCA4b proteins. Thus, the maintenance of a full-length open reading frame of the *CLCA4b* gene in the GenBank database sequences as well as its slight but specific evolutionary differences suggest functional adaptation as a result of the genetic duplication.

**Fig 2:**
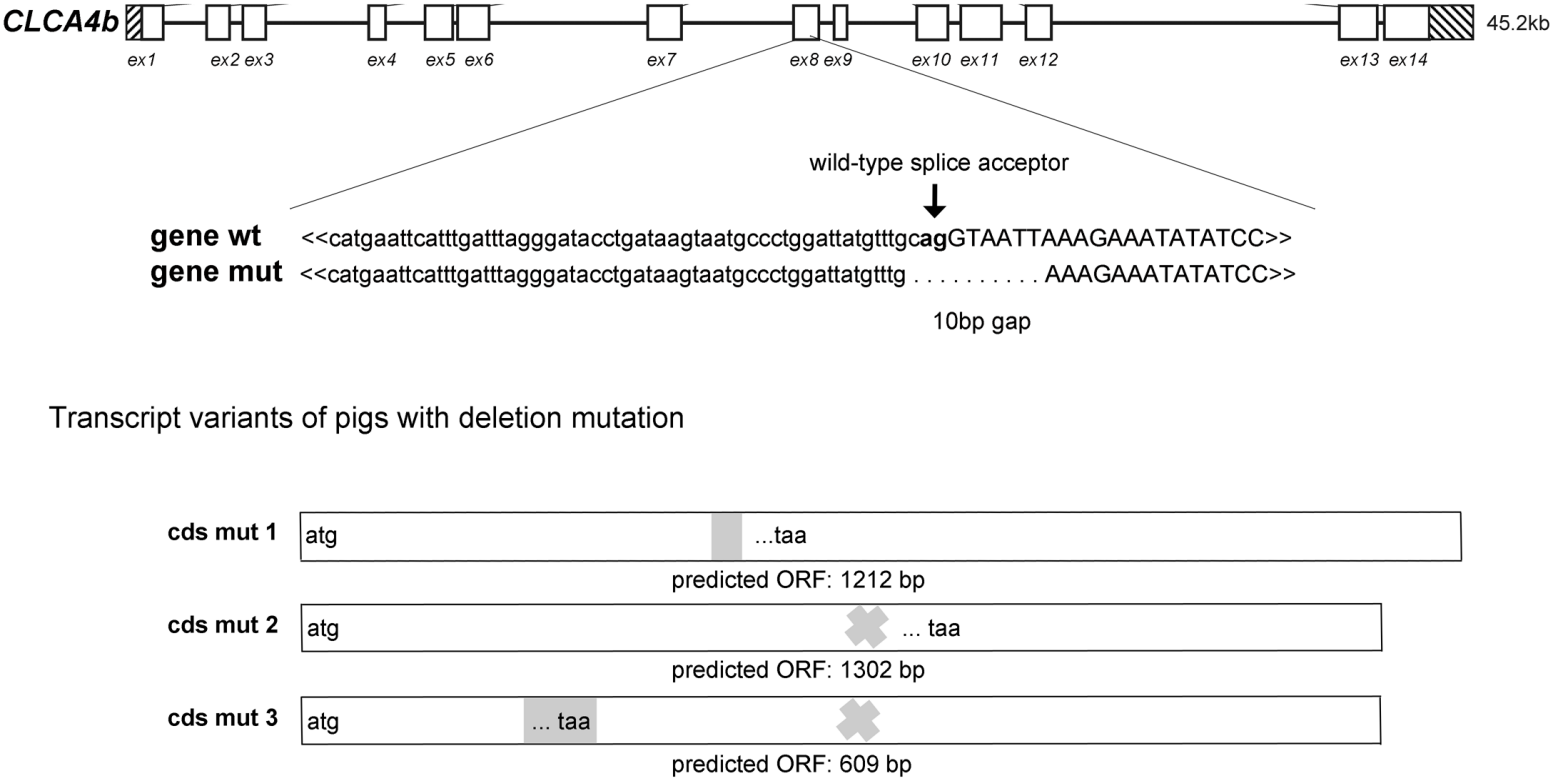
A deletion mutation in the *CLCA4b* gene leads to transcripts with a premature termination codon. A 10 base pair (bp) deletion was found at the splice acceptor site of exon (ex) 8. The mutated gene (mut) coded for different alternatively spliced mRNA species (cd smut 1 to 3) with an insertion (grey box), loss of exon 8 (grey cross) or the insertion (grey box) and loss of exon 8 (grey cross). All alternatively spliced transcripts had a premature termination codon (PTC) with shortened predicted ORFs.

In contrast to these findings, however, there was occasional evidence of a 10 bp deletion in *CLCA4b*, involving the 5´-end of exon 8 as well as the upstream intronic region (Fig 2), resulting in the loss of the splice site acceptor site of intron 7. As a consequence, variations of alternative splicing of the *CLCA4b* transcripts were observed in pigs bearing this naturally occurring mutation (Fig 2). While transcripts from wild-type *CLCA4b* showed the predicted nucleotide sequence with a coding sequence (cds) of 2,766 bp, alternative splicing to an AG-site 38 bp upstream of exon 8 resulted in an insertion of 28 bp into the transcript that causes a frame shift and generates a premature termination codon (PTC) within exon 8 (Fig 2, cds mut 1). Alternatively, splicing out exon 8 (178bp) resulted in a frame shift and a PTC within exon 9 (Fig 2, cds mut 2). Occasionally, outsplicing of exon 8 concurrently occurred with an alternative splicing to exon 4, introducing a sequence of 105 bp to the cds (Fig 2, cds mut 3), and again causing a deleterious modification of the transcript. However, such transcript variant was only found in a single individual and we did not further examine whether this finding was specific for the mutated variant or a common effect of *CLCA4b*.

We then attempted to estimate the prevalence of the 10 bp genomic deletion in the porcine population and in certain breeds. Genomic DNA of various pure- and mixed-breed pigs was analyzed using the restriction endonucleases *Dra*I and *Tsp*509I to distinguish between mutated, wild type and heterozygous individuals. In a total of 103 pigs, we found incidence for underrepresentation of wild-type alleles (16.5%) compared to pigs bearing the mutation on both alleles (39.8%) or on a single allele (43.7%; Table 3), but the dominance of pigs from commercial meat production units might have biased this statistical analysis. More specifically, we identified breeds that either lacked the wild-type allele (Duroc) or the mutated allele (Large White, Cerdo Iberico), but the low number of animals from those breeds compromises statistically reliable conclusions.

**Table 3:** Incidence of *CLCA4b* wild type, mutant and heterozygous pigs in various porcine breeds.

Breed	No. of individuals	Wild type	Mutant	Heterozygous
Mangalitsa	4	2	2	-
Hampshire	3	-	-	3
Large White	3	3	-	-
Cerdo Iberico	3	3	-	-
Pietrain	3	-	1	2
Duroc	3	-	3	-
Swabian	3	1	-	2
Large Black	3	2	-	1
German Landrace	5	2	1	2
Turopolja	3	-	1	2
Mixed breed (EUROC x Pietrain)	5	1	1	3
Mixed breed (unknown cross-breeding)	65	3	32	30
Total	103	17 (16.5%)	41 (39.8%)	45 (43.7%)

### Generation and specificity of anti-*CLCA4b* antibodies

**Fig 3:**
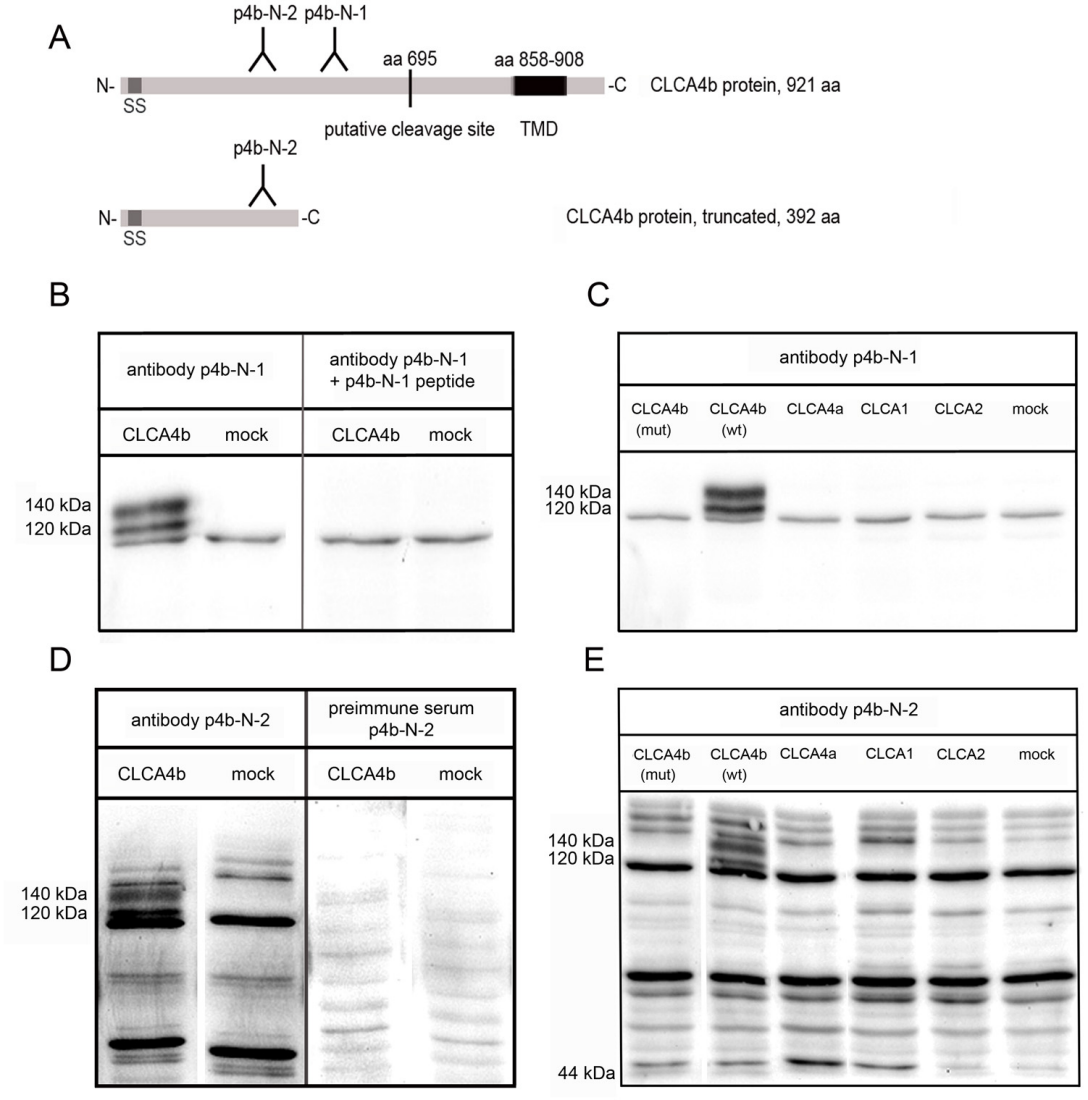
Both antibodies against the amino-terminus of *CLCA4b* detect two specific bands in *CLCA4b* transfected cell lysate. (A) Two antibodies against CLCA4b were generated, both against peptides corresponding to the proposed amino-terminal cleavage product of the protein. The length and mass of the theoretically truncated protein CLCA4b were calculated, and antibody p4b-N-2 was designed to also recognize a truncated CLCA4b protein in the immunoblot. SS = signal sequence; TMD = transmembrane domain; C = carboxy-terminus; N = amino-terminus. (B) Antibody p4b-N-1 recognized an approx. 140 kDa precursor protein as well as a 120 kDa amino-terminal cleavage product in the cell lysate of HEK293 cells transiently transfected with the CLCA4b ORF in pcDNA3.1 (left). No specific bands were detected when cells were transfected with the pcDNA3.1 vector alone (mock). Preabsorption of antibody p4b-N-1 using 20 μg/ml of its specific peptide resulted in complete abolishment of the specific bands (right pattern). (C) Antibody p4b-N-1 failed to detect any specific bands in cell lysates of cells transfected with other porcine CLCA family members, CLCA1, CLCA2 or CLCA4b. In cells transfected with the CLCA4b clone carrying the additional intron sequence due to the genomic deletion (CLCA4b mut), no specific protein bands were detected, arguing for a complete inhibition of protein translation in these cells. In comparison, both the precursor protein and the amino-terminal cleavage product are clearly visible when cells were transfected with the intact CLCA4b clone without a deletion (CLCA4b wt). (D) The results of the antibody p4b-N-1 were confirmed by antibody p4b-N-2 which recognized two band of the same size in cell lysates from HEK293 cells transiently transfected with the CLCA4b ORF in pcDNA3.1 (left pattern). No specific bands were detected in lysates from mock transfected cells. Preimmune serum served as negative control and failed to reveal any specific bands (right pattern). (E) No specific bands were detected by antibody p4b-N-2 when cells transfected with CLCA1, CLCA2, or CLCA4a clones were subjected to immunoblotting. Interestingly, although this antibody would be capable of detecting a possibly shortened CLCA4b protein, no specific bands were detected in cell lysates from cells transfected with the CLCA4b (mut) clone, arguing for a complete translation stop of the CLCA4b protein in case of the mutation.

*In silico* analyses of the CLCA4b amino acid sequence using several prediction programs suggested a transmembrane domain between aa 882 and 908, with a carboxy-terminal intracellular domain and an amino-terminal extracellular tail (Fig 3A).

Total cell lysates from pCLCA4b-transfected HEK293 cells or whole intestinal tissue lysates were immunoblotted with antibody p4b-N-1 diluted 1:5,000. The antibody clearly recognized a 140 kDa protein, consistent with the canonical CLCA-precursor protein, and a 120 kDa protein, consistent with its amino-terminal cleavage product (Fig 3B). Both bands were absent when the preimmune serum was used. Moreover, the two bands disappeared when the antibody was preabsorbed with its specific peptide (Fig 3B). Preincubation of the antibody with an irrelevant peptide instead of the specific peptide in the same concentration revealed no changes in signal intensity. Cells transfected with the pcDNA3.1 vector alone (mock) served as negative control. Thus, the CLCA4b protein was processed according to the well-characterized path of other CLCA proteins, including post-translational cleavage of the precursor protein [20].

Antibody specificity was further confirmed by immunoblotting analysis of HEK293 cell that have been transfected with expression constructs of any other known porcine CLCA members (CLCA1, CLCA2, CLCA4a). Whereas CLCA proteins were successfully detected by using the respective antibodies, neither p4b-N-1 nor p4b-N-2 did detect specific bands in any of those cases (Fig 3C and 3E).

In contrast to the wild-type CLCA4b, no specific bands were detected when lysates of HEK293 cells transfected with a vector expressing the mutated form of pCLCA4b (Fig 3C). This was particularly true for antibody p4b-N-2 that was designed to also detect the truncated form of CLCA4b (Fig 3D and 3E).

### Tissue expression pattern of the *CLCA4b* protein and phenotyping of *CLCA4b* mutant pigs

**Fig 4:**
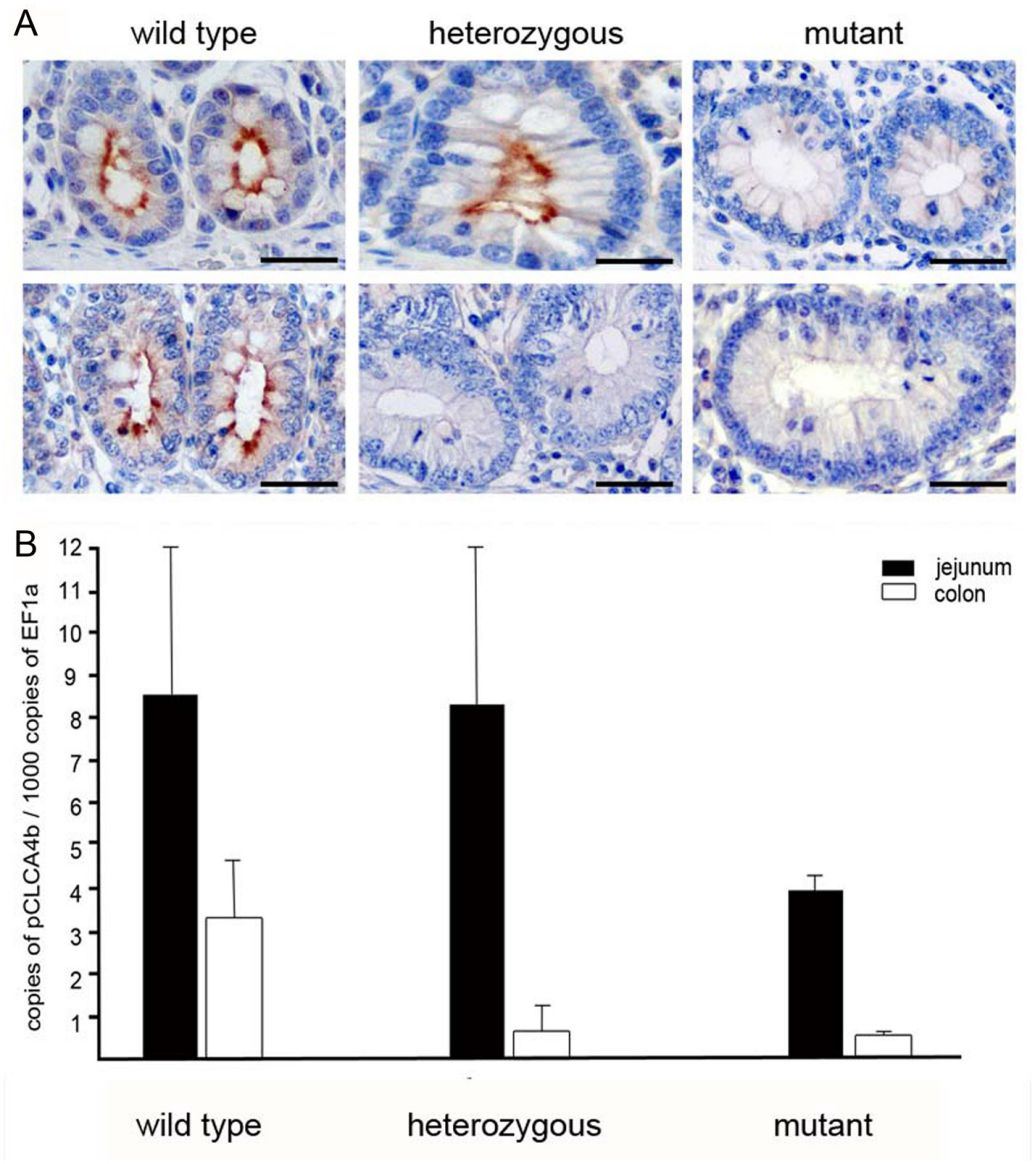
The *CLCA4b* protein is exclusively located in apical membranes of intestinal crypt epithelial cells and shows high variations in response to the genomic deletion of *CLCA4b*. (A) In wild type pigs without the genomic deletion of CLCA4b, the protein is invariably located at apical membranes of non-goblet and goblet cells in the crypts of the small (upper left) and large (lower left) intestines. In heterozygous pigs, the protein is similarly distributed in the small intestine (upper central) whereas it is completely absent from the large intestine (lower central). In pigs with the genomic deletion of CLCA4b, no CLCA4b protein was detected, neither in the small (upper right) nor in the large intestine (lower right). Immunohistochemistry using antibody CLCA4b-N-1 diluted 1:8,000. Bars: 80 μm. (B) Quantitative RT-PCR expression analysis from jejunum (black) and colon tissues (white) revealed CLCA4b expression twice as high in the jejunum than in the colon of CLCA4b wild type pigs (left). When compared to wild type pigs, CLCA4b mutant pigs (right pattern) had a markedly decreased amount of CLCA4b mRNA in both the jejunum and colon. While CLCA4b mRNA copy numbers were also reduced in the colon of heterozygous compared to wild type pigs, copy numbers of CLCA4b in the jejunum were markedly increased (central pattern). Results are mean +/- SEM.

To elucidate the tissue and cellular expression pattern of CLCA4b, antibody p4b-N-1 was used for immunohistochemical protein localization in various formalin-fixed, paraffin-embedded tissues. In pigs carrying the wild-type form of the gene, the CLCA4b protein was exclusively detected in apical membranes of small and large intestinal crypt epithelial cells (Fig 4A) but not in any other tissue. The specificity of this signal was confirmed by pre-incubation of the antibody with its specific peptide and the use of the preimmune serum which both resulted in complete loss or strong reduction of the signal.

In animals bearing the mutated form of the *CLCA4b* gene on both alleles, protein was neither found in the small nor in the large intestine nor in any other organ (Fig 4A). Remarkably, in heterozygous animals, we found a strong and consistent CLCA4b protein signal in the small intestine (Fig 4A), but the protein was fully absent in any of the large intestinal compartments.

Immunohistochemical and histopathological analyses of intestinal tissues of homozygous wild type (n = 2) and homozygous mutant pigs (n = 4) did not indicate any differences in CLCA1, -2 or -4a protein expression or any structural or pathological differences as a result of the *CLCA4b* genotype.

The mRNA expression pattern investigated by conventional RT-PCR confirmed these results, showing strong and consistent specific signals for the intestinal tract only. Weak signals of the *CLCAb* transcripts were obtained from the upper respiratory tract and from the eye whereas their protein was neither detected by immunohistochemistry nor by immunoblotting in these tissues. Quantitative RT-PCR revealed that jejunal and colonic expression levels of *CLCA4b* mRNA in mutant pigs were approximately half (jejunum) and one third (colon) of its expression in wild type pigs (Fig 4B). In correlation to the findings by immunohistochemistry, heterozygous pigs showed similar expression levels in the jejunum as compared to wild type, whereas the CLCA4b expression in the colon was approximately three times lower compared to wild type pigs.

### *CLCA4b* does not evoke a calcium-dependent chloride conductance in HEK293 cells

**Fig 5:**
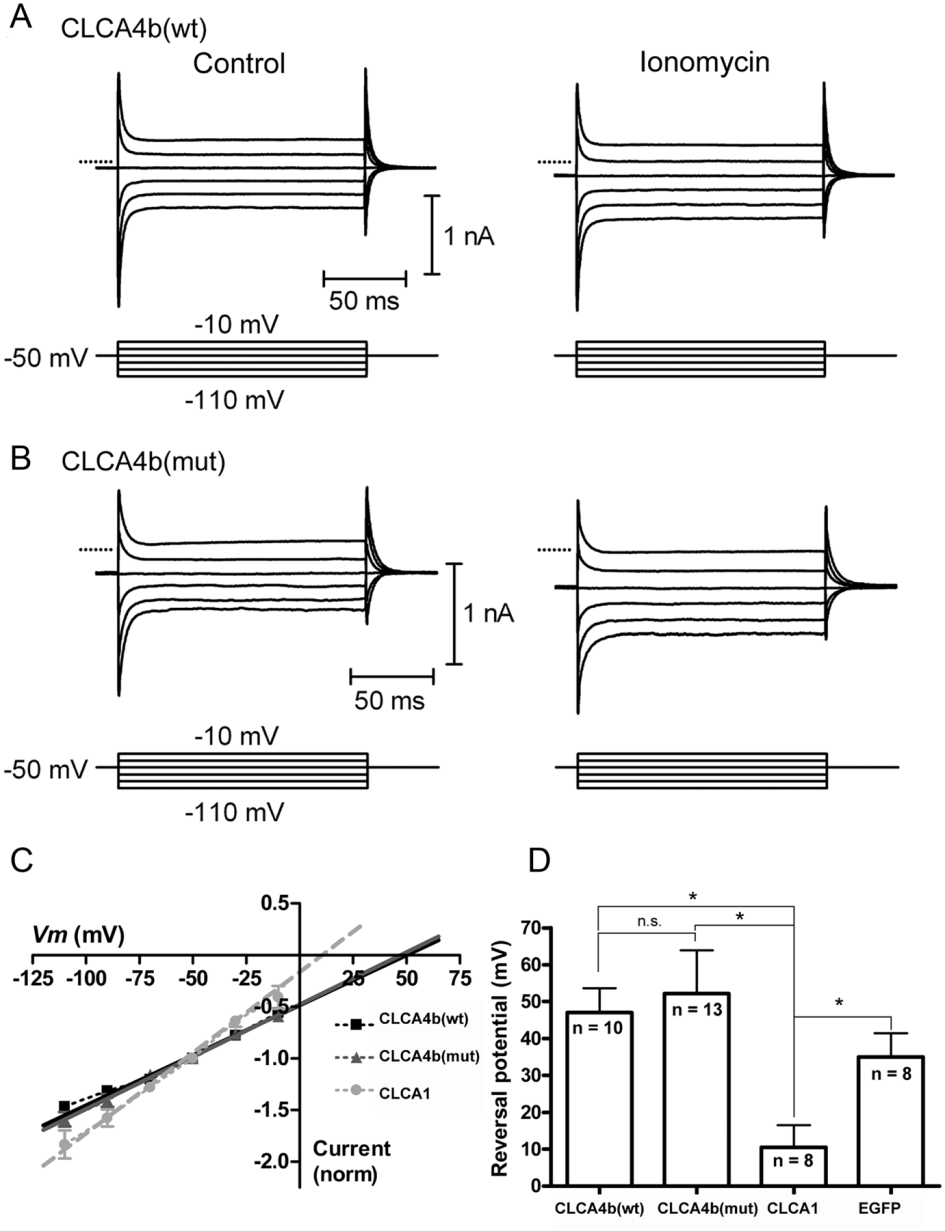
*CLCA4b* does not evoke a calcium-dependent chloride current characteristic of other *CLCA* family members. (A) Currents were evoked in HEK293 cells expressing CLCA4b (wt) by a series of voltage steps (150 ms, -110 to -10 mV) before (left) and after (right) application of the calcium ionophore ionomycin (10 μM). Ionomycin stimulated an inward current at the holding potential of -50 mV and increased the amplitude of step-evoked currents. (B) Similar experiment for HEK293 cells expressing CLCA4b (mut). In A and B, zero current is indicated by the dotted lines. (C) Current-voltage relationship for the difference currents were obtained by subtracting the step-evoked currents before from those after ionomycin application. Linear extrapolation was used to determine the reversal potential (E_rev_) for the ionomycin-stimulated currents which were normalized to the current at the holding potential (-50 mV). (D) Mean E_rev_ for ionomycin-stimulated currents in cells expressing CLCA4b (wt) (n = 10), CLCA4b (mut) (n = 13), CLCA1 (n = 8), serving as a positive control, or EGFP alone (n = 8), which served as a negative control. *p < 0.005, n.s. p > 0.05, independent t-test.

Whole-cell recordings were obtained from CLCA4b-expressing HEK293 cells as confirmed by EGFP or RFP fluorescence (Fig 5). Shortly after application of the calcium ionophore ionomycin (10 μM), we observed a slowly-developing inward current. In cells transfected with cDNA coding for CLCA4b (wt) or CLCA4b (mut), this current reversed near the estimated equilibrium potential for calcium (Ca^2+^) (CLCA4b wt: +47 ± 6 mV, n = 10; CLCA4b mut: +52 ± 12 mV, n = 13; Fig 5C and 5D). In HEK cells transfected with EGFP alone, the ionomycin-evoked current reversed at +35 ± 6 mV (n = 8) which did not differ significantly from CLCA4b (wt)- or CLCA4b (mut)-transfected cells (p > 0.05). The inward current under these conditions is likely the result of the Ca^2+^ conductance created by insertion of the ionomycin ionophore into the membrane, suggesting that elevated intracellular [Ca^2+^] did not trigger a chloride (Cl^-^) conductance in cells expressing CLCA4b (wt), CLCA4b (mut) or EGFP alone. In contrast, we successfully recorded ionomycin-evoked anion currents in cells transfected with cDNA for CLCA1, a member of the CLCA family known to induce a calcium-activated chloride conductance [32]. As with cells transfected with CLCA4b (wt) or CLCA4b (mut), application of ionomycin was followed by an inward current, however, this current reversed at 11 ± 6 mV (n = 8; Fig 5D) and developed more quickly. This reversal potential was significantly less positive than that for CLCA4b (wt) (p<0.005, independent t-test) or for CLCA4b (mut) (p<0.01, independent t-test), and fell between the equilibrium potentials for Ca^2+^ and Cl^-^, suggesting that elevated intracellular [Ca^2+^] triggered a Cl^-^ conductance in cells expressing the equine CLCA1, consistent with its previous characterization [34].

## Discussion

The recently available CF pigs are regarded as the most suitable animal models because they mimic the human disease much more closely than the murine models do [22–27]. Members of the CLCA gene family, particularly CLCA1 and CLCA4, have been shown to modulate several aspects of the human CF phenotype [1,3,4]. Interestingly, significant differences have been described between several human CLCA and their orthologous murine genes and proteins, suggesting further difficulties in translating phenotypical features of murine CF models to the human disease. For example, the human *CLCA* gene locus consists of only four genes, whereas the murine gene locus consists of eight genes [20] and the human *CLCA3* is thought to be a pseudogene whereas its murine orthologs are not [21]. Furthermore, the cellular expression pattern of the human CLCA4 is quite different from that of its murine ortholog [17]. Thus, with the generation of CF pig models, the porcine CLCA genes and proteins have become a focus of interest in terms of potentially CF-relevant differences to their human orthologs. Specifically, we have started to systematically characterize the genetic structure, tissue and cellular expression patterns of their porcine CLCA homologs to evaluate their relevance for the phenotype in the CF pig models [28–30].

Unlike in any other mammalian species tested so far, the *CLCA4* gene has undergone a duplication in the pig resulting in an additional gene which we termed *CLCA4b*, located between the parental *CLCA4a* and the *CLCA3* genes on chromosome 4 [28–30]. The results of this study suggest that this apparently pig-specific gene is transcribed into a full length protein with several hallmark characteristics of other CLCA proteins, including its length of 921 amino acids, a conserved amino domain which is thought to function as a metalloprotease [35], the van Willebrand-factor A domain, a domain of unknown function and a predicted single transmembrane domain. Of note, our bioinformatic analyses strongly argue for evolutionary pressure and functional adaptation of the protein.

Among the most relevant results is the obviously highly cell-type specific expression pattern of the CLCA4b protein which is different from that of CLCA4a and also from that of any other CLCA proteins in the pig and other species investigated so far. While CLCA4a is selectively expressed at the apical membranes of enterocytes at the villus tips in the small and large intestine [29], its duplication product CLCA4b was only found at the apical membrane of small and large intestinal crypt epithelial cells. The functional consequence of this separation remains elusive so far.

Similar to its parental gene product CLCA4a [28], CLCA4b covers a broader tissue expression pattern on mRNA than on protein level which has also been reported for other CLCA members, such as the murine Clca2, formerly known as mClca5 [35]. It is unknown to date whether tissue specific posttranscriptional regulatory events preventing protein synthesis or a tissue-dependent low and therefore immunohistochemically undetectable protein level may account for this observation.

**Fig 6:**
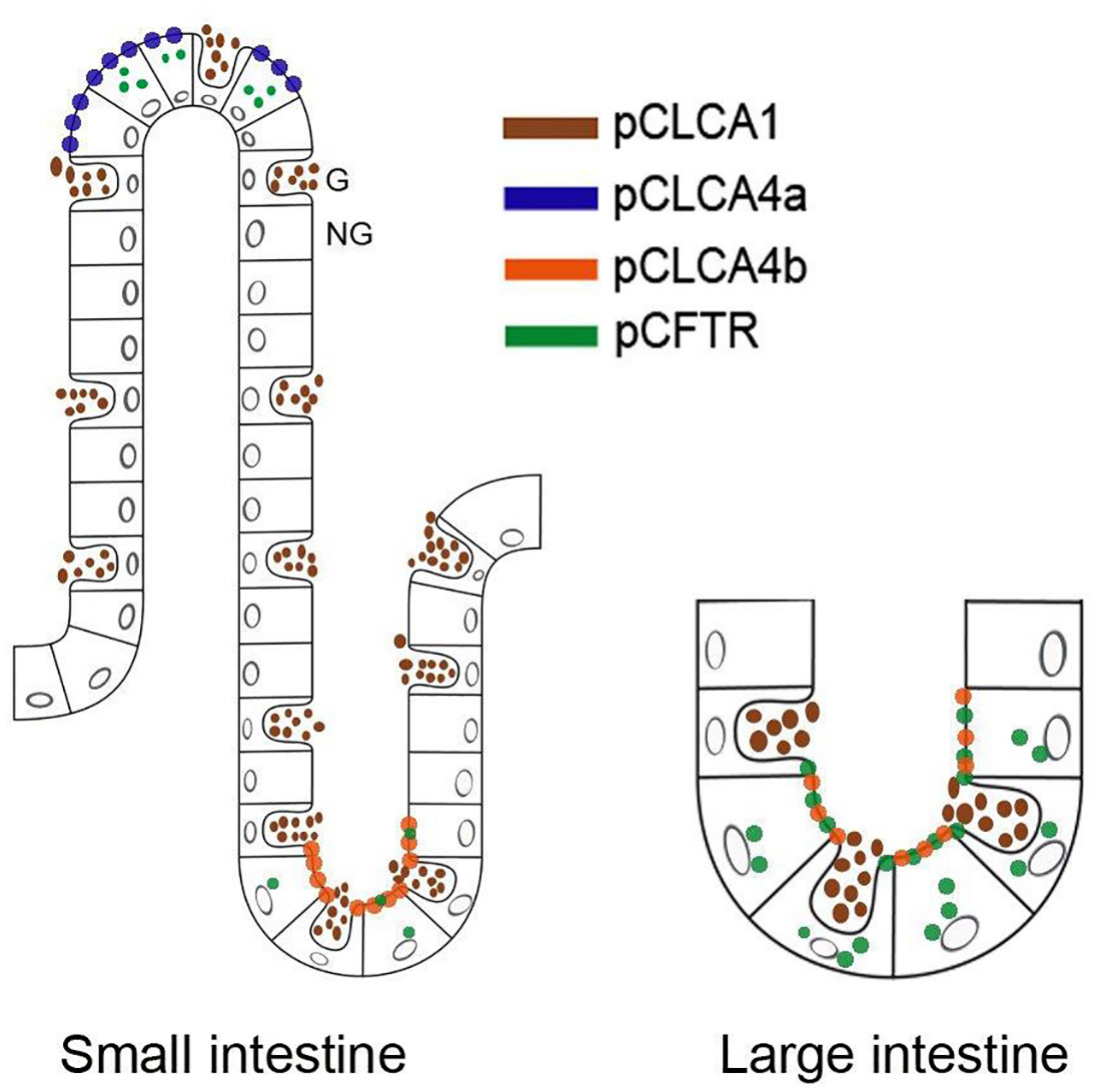
The porcine *CLCA* proteins *CLCA1*, *CLCA4a* and *CLCA4b* occupy different cellular niches in the intestinal tract. The CLCA4a protein (blue) had previously been detected exclusively in small intestinal villus enterocytes. In contrast, its paralog CLCA4b (orange) is expressed only in small and large intestinal crypt epithelial cells where the porcine CFTR protein (green) is also expressed. Moreover, a third CLCA protein, the fully secreted CLCA1 (brown) is expressed by goblet cells. Thus, each porcine CLCA protein appears to occupy a distinct functional niche in small and large intestine. Interestingly, the combination of CLCA4a (blue) and CLCA4b (orange) represent the expression pattern of their single murine ortholog CLCA4a (compare Bothe et al. 2008). G = goblet cell, NG = non-goblet enterocyte.

Interestingly, the murine Clca4a protein, formerly termed mClca6, is expressed in enterocytes both at the villous tips and deep crypts [17], arguing that both microenvironments are served by the same Clca protein in mice. The duplication of the two porcine *CLCA4* genes and the cellular expression of their products in the same tissue environment (Fig 6) thus support the notion of species-specific divergences of *CLCA* genes which has previously been observed in other species and for other members of the CLCA family of proteins [36]. Whether the change of expressing cell type goes along with a similar or changed protein function or regulatory pathways will have to be established in the future. A second obvious difference between the pig *CLCA4a* and *CLCA4b* genes and the mouse Clca4 ortholog clearly lies in their different expressions in the respiratory tract: while the porcine CLCA4a was convincingly detected at the apical membranes of tracheal and bronchial epithelial cells [28], neither the duplicated CLCA4b protein nor the mouse CLCA4 orthologous proteins [17] were found to be expressed in the trachea or lungs. Details on the cellular expression of the human CLCA4 protein have not yet been reported and await further studies. The porcine CLCA1 is another CLCA protein expressed in the intestine which, however, has been found in mucus-producing goblet cells only but not in non-goblet cell enterocytes [27]. The close proximity of the two CLCA4 variants in pigs to this third CLCA protein further supports the notion of different CLCA members covering different cellular and probably functional niches in the intestine [17] (Fig 6).

Several members of the CLCA family in pigs and in other species are capable of modulating calcium-activated anion conductances *in vitro* [36], and the cellular expression patterns on the apical membrane of intestinal (CLCA4a and -b) and respiratory cells (CLCA4a only) support the proposed role in transepithelial anion conductance [29]. Recently, the chloride channel protein TMEM16A has been identified as a molecular target of CLCA1 [16]. Alternative chloride currents which are not mediated by CFTR have been proposed to possibly compensate for the defective chloride secretion in CF patients and are considered promising therapeutic targets [8–10]. In humans, the *CLCA1/CLCA4* gene locus acts as a modifier of rectal anion conductance of CF patients and allelic variants of both CLCA1 and CLCA4 are associated with the severity of intestinal phenotype or the capability to express residual chloride secretion in the colon epithelium, respectively [1,3,4]. Consistently, the murine ortholog to hCLCA4 induces a calcium-activated chloride conductance [18]. Surprisingly, in our study the porcine CLCA4b was unable to evoke a calcium-activated chloride current *in vitro* in this study.

In light of its role as modulator of the CF phenotype in humans [1,3,4], it appears noteworthy that only the porcine CLCA4b but not CLCA4a is expressed by the same crypt epithelial cells that also express the CFTR protein (Fig 6) [33]. Both the separation into the two gene products *CLCA4a* and *CLCA4b* in pigs only with an as yet unknown functional significance and the observation that a large percentage of pigs harbors a complete deletion of the CLCA4b protein expression give ample reason to assume that its alleged modulatory role may differ between pig models of CF on one side and mouse and human CF on the other. A detailed characterization of the functions of the CLCA4 variants and the consequence of the complete loss of CLCA4b in the context of the three available pig models of CF [22,23,26] will realistically be required before the CF phenotype in pigs can be fully interpreted to better understand the human disease. In contrast to the situation in humans with 15% of CF neonates developing meconium ileus, 100% of CF piglets present this lesion, along with an overall worse intestinal phenotype [22,23,26]. It will be interesting to explore whether the porcine-specific CLCA4 variants or the mutation of CLCA4b have an impact on this striking intestinal phenotype in pigs and may contribute to this noteworthy difference to human CF.

In addition to their well-established induction of ion conductance, some CLCA proteins have functionally been linked to the induction of expression of mucus genes [20] or cytokines [37,38] as well as cellular differentiation [39,40]. The human CLCA4 protein, for example, seems to a play a role in epithelial differentiation since its loss promotes epithelial-to-mesenchymal transition *in vitro* [40]. Thus, the unique cellular expression pattern of CLCA4b in crypt epithelial cells raises the question of whether it may serve a related function in cellular differentiation processes since crypts are the niche of stem cells for intestinal epithelial cell renewal and differentiation.

In this study, we revealed a unique *CLCA4b* gene mutation resulting in complete loss of the protein in a remarkably large subpopulation of pigs. The 10 bp deletion causes alternative splicing, generating distinct transcripts of the CLCA4b mRNA which invariably result in a premature termination codon as result of a frameshift mutation. It is plausible that nonsense-mediated decay [41–43] prevented the synthesis of a truncated, putatively nonfunctional protein, an assumption that was well confirmed when we failed to detect any truncated protein in immunoblot analysis of HEK293 cells transfected with the mutated form of *CLCA4b*.

In our test cohort of more than 100 pigs, one or two mutated and thus non-functional alleles were found in ancient as well as modern pig breeds. Although we failed to observe any clinical or histological phenotype in the affected animals, this does not necessarily mean that the gene function is ultimately dispensable for pigs, as a specific phenotype might only appear under specific challenges. The high prevalence of the mutation in all pig breeds tested suggests that the mutation has occurred before modern pig breeds arose or that the mutation has been distributed among pig breeds by systematic cross-mating. The latter might, in turn, imply that the mutation of *CLCA4b* (or a yet undefined allele variant in close vicinity to the *CLCA4b* gene) has a selective advantage over the wild type form that resulted in a preferential selection for breeding purposes. Although the biological basis for such an evolutionary trait is completely unclear, this is not untypical for the evolution of lifestock animals and it is also in correlation to genetic defects in human beings, where heterozygous carriers of sickle cell anemia alleles have a selective advantage in regions with persistent malaria infections. Also, the expansion of mutated CFTR alleles in the human population have been repeatedly considered as a result of the higher resistance of heterozygous carriers to secretory diarrhea when intestinal pandemies caused major losses on the central European population in the Middle Ages [44]. Additionally, the natural knockouts and possibly even the heterozygous carriers may serve as models to study the actual function of the CLCA4 protein and the pathways it is involved in, similar to the use of experimental mouse knockout models in terms of a functional genomics approach.

In summary, the pig-specific duplication of the *CLCA4* gene results in two functional proteins, CLCA4a and CLCA4b, which have segregated into two adjacent cellular niches, i.e. the intestinal epithelial cells at the villous tips versus those deep in the crypts. In the mouse and likely other mammalian species, both cell types express the protein product of the unduplicated parent *Clca4* gene. The functions of the two CLCA4 protein variants in pigs and the biomedical consequences of their separation, specifically in terms of their alleged modulatory role in CF, remain to be established. Surprisingly, a *CLCA4b* gene mutation resulting in the complete loss of protein expression in homozygously affected animals is persistent in the pig population. This mutation seems to have no impact on any phenotype at first sight. These CLCA4b-specific features will have to be considered in future studies using any of the pig models of CF, specifically in light of the putative modulatory role of CLCA4 in CF.
